# A Simplified Model for the Study of Film-Boiling Droplet Motion on Microscale Ratchets

**DOI:** 10.3390/applmech5010006

**Published:** 2024-01-30

**Authors:** Sheldon Wang, Jeong Tae Ok, Sunggook Park, Mahmoud Elsharafi, Yu Guo

**Affiliations:** 1 McCoy College of Science, Mathematics & Engineering, Midwestern State University, a Member of the Texas Tech University System, Wichita Falls, TX 76308, USA;; 2 Electromechanical Engineering Technology, Shawnee State University, 940 Second Street, Portsmouth, OH 45662, USA;; 3 Department of Mechanical & Industrial Engineering, Louisiana State University, 3290M Patrick F. Taylor Hall, Baton Rouge, LA 70803, USA;

**Keywords:** Leidenfrost, ratchet, droplet, computational fluid dynamics, meshing, vapor, mass transfer, heat transfer

## Abstract

In this work, we explore a simplified model based on both analytical and computational methods for the study of film-boiling droplet motion on microscale ratchets. We consider a specific ratchet design with the length periods and depth of ratchets much smaller than the size of the droplet. We conclude based on our modeling that for the ratchet configuration considered in this paper, the conduction within the vapor film is the dominant means of heat transfer in comparison with convection and radiation. Furthermore, we demonstrate a more manageable two-dimensional model in which analytical approaches coupled with computational approaches yield reasonably accurate results in comparison to the actual experiments.

## 1. Background and Research Scopes

Leidenfrost effects refer to the self-levitation phenomenon of liquid droplets on a solid surface at a temperature sufficiently higher than the evaporation or boiling temperature of the liquid. It is easy to imagine that a perfect symmetric droplet could be levitated with the flux of evaporating liquid and its linear momentum in the gravitational direction. However, a slight imperfection or different surface morphology will trigger a horizontal motion since the self-levitation dramatically reduces the resistance in this direction. Moreover, a strategically created ratchet, a surface contour with spatial periodicity with asymmetric structures, has recently received much attention as a means of rectifying motion in the absence of net force. This “net force-free” large-scale motion produced by ratchets is actually due to local asymmetric forces that are averaged to be zero over space and time. Ratchets have been used for some applications including quantum-tunneling ratchets [1], particle separation via Brownian ratchets, cell separation by microfluidic funnel ratchets [2], dielectrophoretic rectification Brownian motion [3], and the action of molecular motors [4]. A decade ago, the self-propelled directional motion of liquid droplet [5] was achieved by combining a nonpermeable solid asymmetric ratchet (period: 1.5 mm and depth: 0.3 mm) and the liquid’s Leidenfrost (or film-boiling) regime [6]. A liquid droplet generally floats on a thin continuous vapor film in the Leidenfrost regime thanks to the poor heat transfer via the film between the solid and liquid. As the liquid evaporates at the bottom surface of the droplet, the pressure that levitates the droplet pushes out the vapor laterally and the ratchet’s surface partially rectifies this vapor flow, exerting a net viscous force (or viscous drag) on the droplet. Linke et al. observed a droplet velocity of a few centimeters per second with the millimeter scale of ratchets in the range of droplet volume 50≃200μL (radii ≫ capillary length) [5]. By following Linke’s work, there have been some studies on theories and hypotheses that provide a certain level of understanding and explanation of the mechanism related to the motion of the Leidenfrost ratchets system. The Quéré group confirmed the viscous drag mechanism proposed by Linke et al. by observing the self-propelled motion of Leidenfrost solids on a metallic macroscale of the ratchets [7–9]. The Park group have also furthered the investigation by extending this principle of the directional motion of a liquid droplet to micro- and sub-microscale ratchets by releasing the laterally circular shape of droplets (radii < capillary length) [10,11]. They claimed that the ratchets scaling topology had a significant impact on the kinematic quantities of the droplet depending on the kind of liquid and the temperature gradient between the solid and liquid. The viscous drag mechanism proposed by both the Linke and Quéré groups was extended to capillary droplets on the microscale of ratchets [12] and ratchet traps with concentric circular ridges [13]. Li et al. also supported the viscous drag mechanism by the numerical investigation using a thermal multiphase lattice Boltzmann model with liquid–vapor phase change [14]. It has also been hypothesized that the Marangoni effect [15] or thermal creep [16,17] could be one of the partial impacts on driving mechanisms behind this droplet mobility. The alternated directional motion of the Leidenfrost droplet was observed on micro/nanoscale periodic asymmetric pillar arrays and angled fish scalelike self-assembled micro/nanostructures [18,19]. The enhanced water repellency significantly reduced the threshold temperature for the droplet dislocation [20,21]. Also, an enhanced way to manipulate water droplets on three different lateral shapes of asymmetric saw-teeth topography was introduced by using the sound of boiling droplet [22]. However, the driving mechanisms of this self-propelled motion are still under debate and only a few computational modeling studies have been done so far to our knowledge [23,24].

The purpose of this study was to investigate and analyze the driving mechanism behind the Leidenfrost ratchet system on a miniaturized scale via systematic computational and modeling procedures. In this study, through careful parametric analysis, the main force for propulsion and levitation is established as evaporation due to thermal conduction within the thin film of air and vapor mixture. The terminal velocity of the droplet is estimated with the consideration of the viscous skin friction of the top of the droplet, the form drag of the cross section of the droplet, and the viscous shear between the moving droplet and the surface with the considered dimensions of microscale ratchets’ geometry balanced by the gravity and the propulsion due to the evaporation.

## 2. Simplified Model

Before we get started with the ratchet surface condition, we start with a self-levitated condition with an average clearance to the flat rigid surface $d_{0}$. The levitated liquid droplet is also simplified as a symmetric button shape with a circular bottom cross-section with a radius R. To establish the needed critical Leidenfrost temperature $T_{s}$, we assume the mass flow rate through the entire circular cross section due to evaporation $\dot{m}$ as $\pi R^{2}\rho_{g}v_{0}$, where $v_{0}$ stands for the average vapor velocity discharged from the bottom surface of the droplet. Naturally, during evaporation, under the atmospheric pressure, the droplet temperature remains as the saturated temperature $T_{o}$. Therefore, the thermal condition introduces a heat flux $k\left(T_{s}-T_{o}\right)/d_{0}$, where k stands for the thermal conductivity of the vapor. Notice that the physical intuition based on the bulk air (vapor) flow direction suggests that the convection effect is not as significant as the conduction effect, which is validated with the simplified model in Equations (5) and (6). Based on the mass conservation and the energy conservation, we have the following important governing equation

(1) $$k\left(T_{s}-T_{o}\right)=\epsilon_{c}d_{o}v_{o}\rho_{g}h_{fg},$$

where $\epsilon_{c}$ stands for a coefficient as a measure of the contribution of the heat flux due to conduction to the evaporation of the saturated liquid surface of the droplet, $\rho_{g}$ represents the saturated gas (vapor in this case) density, and $h_{fg}$ stands for the latent heat at the atmospheric pressure.

Note that Equation (1) is indeed identical to Equation (5) in Ref. [24] with a slight adjustment of the geometrical parameter. Furthermore, based on the levitation condition, the self-weight of the button-shape liquid must be balanced by the change in the linear momentum in the gravitational direction, namely,

(2) $$\epsilon_{m}\rho_{f}Hg=v_{o}^{2}\rho_{g},$$

where $v_{0}$ refers to the discharging velocity of the vapor, $\epsilon_{m}$ stands for a coefficient as a measure of the weight proportion carried by the vertical change in the linear momentum, g is the gravitational constant, H represents the average height of the button-shape droplet, and $\rho_{f}$ is the saturated liquid density at the atmospheric pressure.

Combining Equations (1) and (2), we can easily establish the following critical temperature condition $T_{s}$ for Leidenfrost effects

(3) $$T_{s}=T_{o}+\epsilon_{c}\sqrt{\epsilon_{m}}d_{o}L\sqrt{\rho_{f}\rho_{g}gH}/k.$$

Using the steam table, at the atmospheric pressure, we have the saturated temperature $T_{o}=100^{\circ }C,\rho_{g}=0.5975\;kg/m^{3},\rho_{f}=958.39\;kg/m^{3},$ and $L\;=h_{fg}=2257\;kJ/kg$. At the atmospheric pressure, we typically have the thermal conductivity of the vapor k=25.1mW/Km. Therefore, we have the following parametric relationship to compare with the existing literature and experimental evidence

(4) $$T_{s}=100+213\epsilon_{c}\sqrt{\epsilon_{m}}d_{o}\sqrt{H},$$

where $d_{o}$ is the clearance in micrometer (μm) and H is the droplet height in millimeter (mm).

**Figure 1.**
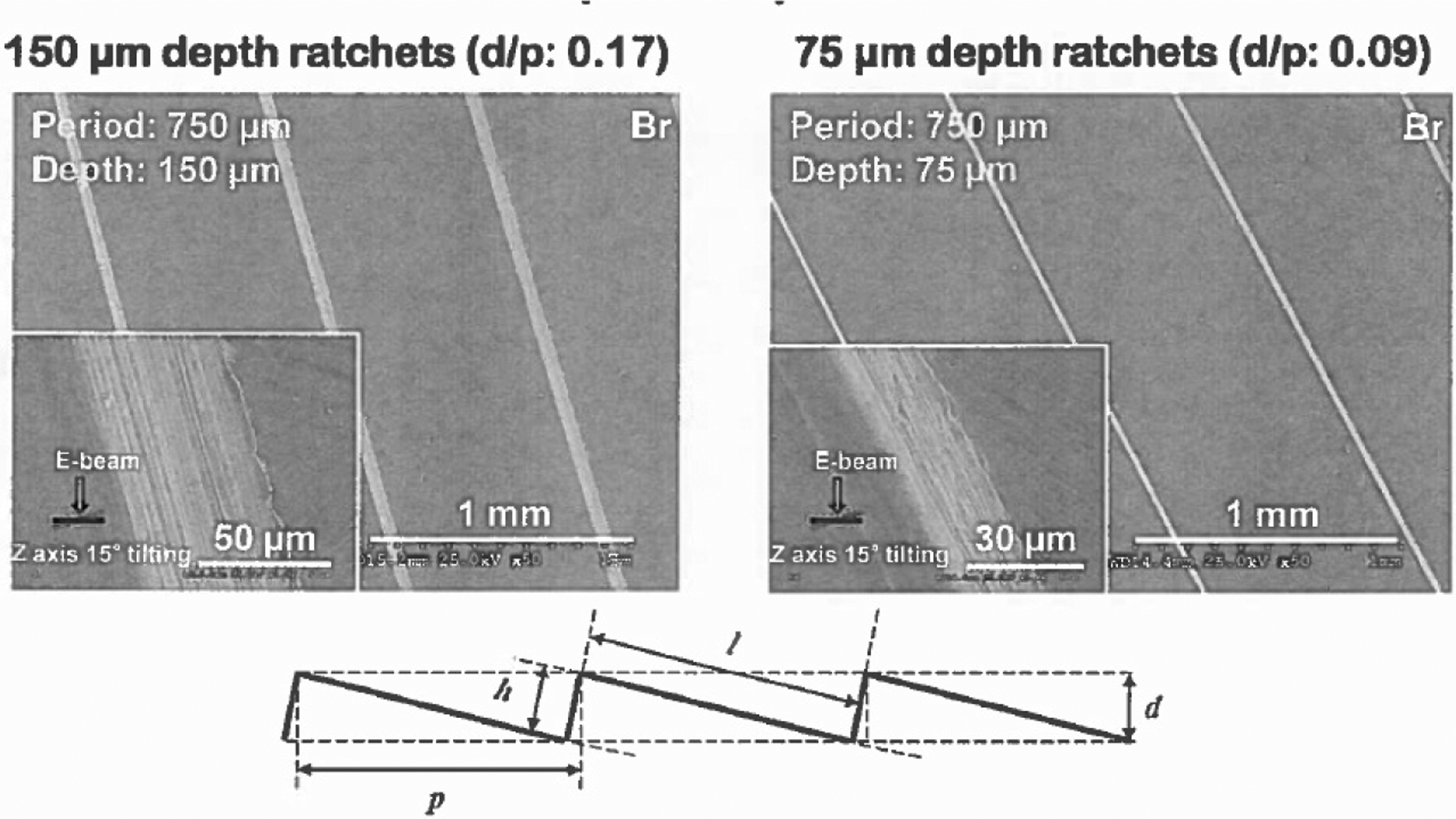
Ratchet surface with key dimensions.

According to the literature [25], the radius of the droplet with a 5-microliter (μL) volume is around 1 mm. Therefore, we can easily assume H to be around 1 mm. Theoretically speaking, if the coefficients $\epsilon_{c}$ and $\epsilon_{m}$ are around the order 1, the levitation clearance $d_{o}$ should be around 1μm to match the empirical evidence of the Leidenfrost temperature around 300 °C [25]. Notice that the proper justification of these two coefficients $\epsilon_{c}$ and $\epsilon_{m}$ can only be derived from a two-dimensional and three-dimensional modeling in which the geometrical effects can be fully explored. In this paper, to further the discussion, we limit our study to the ratchet surface as illustrated in Figure 1, in which the horizontal and incline periods of the ratchet are denoted as p and l, respectively, whereas the vertical and normal clearances are denoted as d and h, respectively. In this study, we assume that h/l and d/p are much less than 1. Assume the droplet spans over N periods of the ratchet teeth; we have Np≃2R, where R is the radius of the curvature of the droplet bottom. In fact, according to Ref. [25], the capillary length a, depicted as $\sqrt{\gamma /\rho_{f}g}$, where γ is the liquid droplet’s surface tension and $\rho_{f}$ is the liquid density, defines the geometry of the droplet with the expression $\delta =R^{3}/a^{2}$, where δ denotes the lowering of the center of mass. This length scale is confirmed for a spherical droplet. The total surface tension lifting the weight of the droplet around the equator of the droplet is γ2πR, and the total weight is approximated by $\rho_{f}g\pi R^{2}h$. Substituting the surface tension γ as $\rho_{f}ga^{2}$ and using a≃R, we can easily have h=2a or h≃2R. For example, for the temperature inside the water droplet to be around the saturation temperature of the water at the atmospheric pressure, namely, 100 °C, the droplet liquid density $\rho_{f}$ is around 960 kg/m^3^ and the surface tension γ is around 59 mN/m. Therefore, the capillary length a is calculated to be 2.5 mm. Moreover, the lowering of the center of mass δ can be denoted as R-h/2, where h is the current thickness of the oval-shape liquid droplet. If the radium of the droplet R is set to be 10 mm, we have the lowering of the center of mass δ estimated to be $\rho_{f}gR^{3}/\gamma$, namely, 2.492 mm. Therefore, according to Ref. [25], the geometric Hertz relation λ calculated with $\sqrt{\delta R}$ is approximated as 2.496 mm, almost identical to the value estimated with $R^{2}/a$, which is 2.496 mm.

Naturally, the radius of the curvature of the droplet depends very much on the surface tension and the pressure difference. We also denote the temperatures at the ratchet surface and the droplet surface as $T_{s}$ and $T_{o}$, respectively. In an atmospheric environment on Earth’s surface, the phase transition temperature for pure water is around 100 °C or 373 K. In this work, the ratchet temperature is around 550 K.

From the derivation of Equation (3) and the empirical evidence of the threshold temperature for Leidenfrost effects, it is feasible for us to utilize a simplified model with the vapor film thickness $d_{o}$ no more than 20μm, much smaller than the normal clearance h for this very complex phenomenon [25], which is essential for the levitation. It is safe to adopt a vapor film thickness or clearance $d_{0}$ between 10μm and 30μm for the hot surface temperature in the range from 200 to 400 °C.

Assuming the normal clearance h is 150μm, the vapor film thickness normal to the ratchet surface $d_{o}$ is approximated as 10μm, whereas the droplet radius is around 1.06 mm. Furthermore, based on Ref. [26], we have the thermal conductivity k as 33 mW/Km for the film of saturated water vapor. Thus, the rate of heat transfer per unit area or heat flux due to thermal conduction within the vapor film between the droplet and the ratchet surface is calculated as follows:

(5) $${\dot{q}}_{\text{cond}}\;≃k\frac{T_{s}-T_{o}}{d_{o}}≃350.5\;kW/m^{2}.$$

Similarly, based on Ref. [27], we have the convective heat transfer coefficient $C_{n}$ as 30 W/Km^2^ for the same film of saturated water vapor. Consequently, the heat flux due to thermal convection within the vapor film is calculated as follows:

(6) $${\dot{q}}_{\text{conv}\;}≃C_{n}\left(T_{s}-T_{o}\right)≃5.3\;kW/m^{2}.$$

We should note that the convective heat transfer coefficient $C_{n}$ depends very much on the air velocity. For the droplet traveling velocity ranges measured in the study of the Leidenfrost effect, the adopted convective heat transfer coefficient is reasonably representative. The Stefan Boltzmann constant for thermal radiation σ is $5.67\times 10^{-8}\;W/m^{2}{\;K}^{4}$. Assuming the emissivity for radiation ϵ is 0.6, we can calculate the heat flux due to thermal radiation as follows:

(7) $${\dot{q}}_{\text{rad}}≃\epsilon \sigma \left(T_{s}^{4}-T_{o}^{4}\right)≃2.5\;kW/m^{2}.$$

Based on the second law of thermodynamics, the direction of the heat transfer in all three means is from the hot ratchet surface to the relatively cold droplet with the saturated temperature at the atmospheric pressure. In fact, the thermal energy required for the phase transition at the surface of the droplet is supplied through the conduction, convection, and radiation heat transfer within the vapor film between the droplet and the ratchet surface as illustrated in Equations (5)–(7). Moreover, it is also clear that the main source of thermal energy is from conduction rather than convection and radiation. Therefore, in the simplified model presented in this paper, we focus exclusively on the thermal conduction within the vapor film.

The lateral dimension of the droplet is of the order of the radius, which is much larger than the typical section or period of the ratchet. Hence, instead of handling a full-fledged three-dimensional droplet hovering on top of many periods of the ratchets, we employ a much simplified two-dimensional generic model to evaluate the propulsion force due to evaporation within each period of the ratchets. This two-dimensional vapor domain is also used to estimate the viscous drag due to the shear of the vapor film. Assuming the droplet travels at a velocity V, the average normal gradient of the vapor film can be estimated as follows:

(8) $$\tau ≃\mu \frac{V}{d_{0}},$$

where the dynamic viscosity of the vapor film μ is roughly 0.00013 Poise with one Poise defined as one dyne per square centimeter multiplied by a second which equals 0.1 Pa ⋅ s.

Of course, this simple estimate was confirmed by a computational fluid dynamics (CFD) calculation of the vapor film between the moving droplet and the stationary ratchet surface. As depicted in Figure 1, the incline of the ratchet surface is defined by an angle β with tan⁡β=h/p=150/764.9=0.1961, which corresponds to the incline angle β equaling 11.1°. For the vapor film, the specific heat $C_{p}$ is averaged as 1.9 kJ/kgK for the temperature range between 373 K and 550 K [28]. Furthermore, during this temperature range, the saturation pressure for water vapor is from 0.1 MPa to 6.0 MPa. Since the atmospheric pressure is constant, the relative humidity within the vapor film can change from 100% to merely 1.7%. This relative humidity is a good indication of the composition of the air within the vapor film, which directly influences the dynamic viscosity and density. In the CFD computation, we employed an average viscosity with a half air and half vapor mixture at the ambient pressure of 0.1 MPa, namely, the density ρ was 1.06 kg/m^3^ and the dynamic viscosity μ was 0.000013 Pa⋅s. It is important to note that the density of moist air is smaller than the density of the corresponding dry air. Moreover, in the full-fledged CFD modeling, the so-called Boussinesq approximation was applied, and the thermal expansion coefficient was adopted at an average temperature of 250° C as 2.21 × 10^−3^ 1/K.

**Figure 2.**
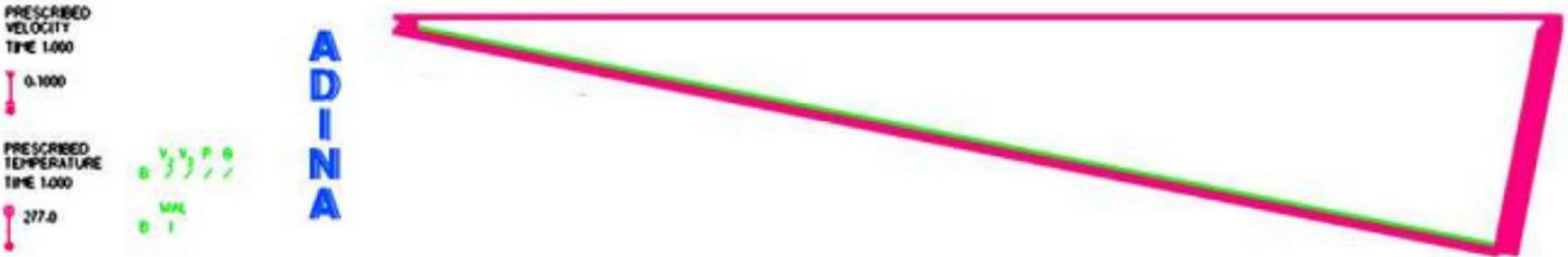
Two-dimensional model of a vapor film for a typical period.

In Figure 2, a two-dimensional computational model is represented. The top surface is moving at a fixed velocity, and the ratchet surface is assumed to be fixed, namely, a wall boundary condition. Moreover, we also adopted a linear velocity profile as the inlet and outlet velocity conditions for a narrow gap between the moving droplet surface and the tip of the ratchets from the moving droplet surface. Similarly, the temperature distribution within the inlet and outlet gap was also linear with the endpoints matching the temperature on the droplet and ratchet surfaces. The illustration of the boundary conditions for fluid dynamics and heat transfer is documented in Figure 2.

**Figure 3.**
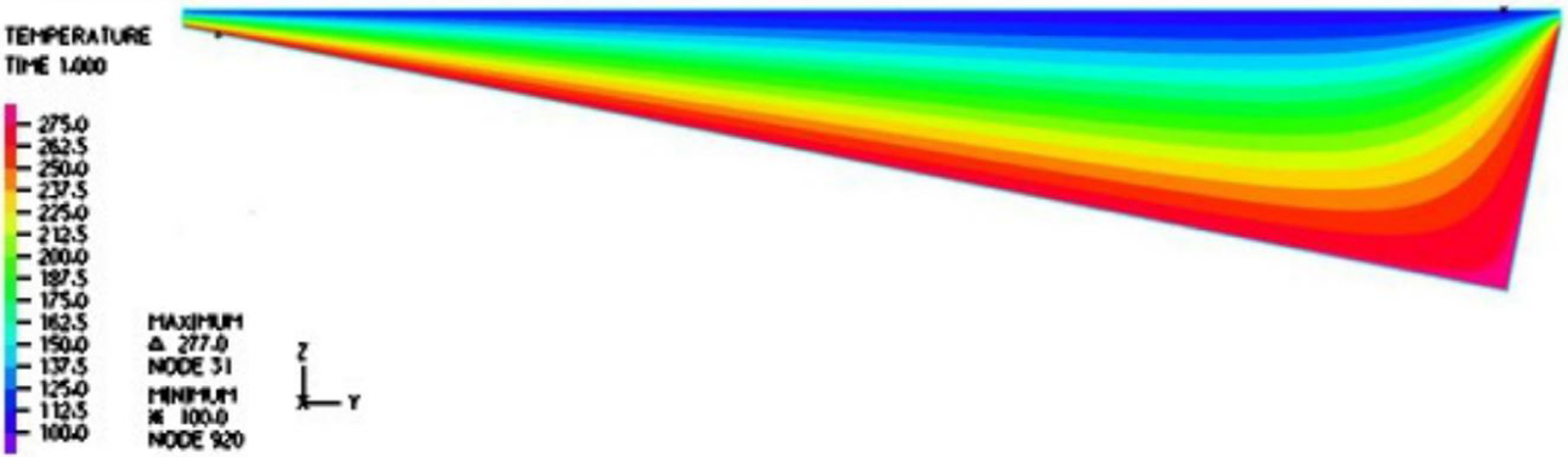
Temperature distribution within the film for a typical period.

**Figure 4.**
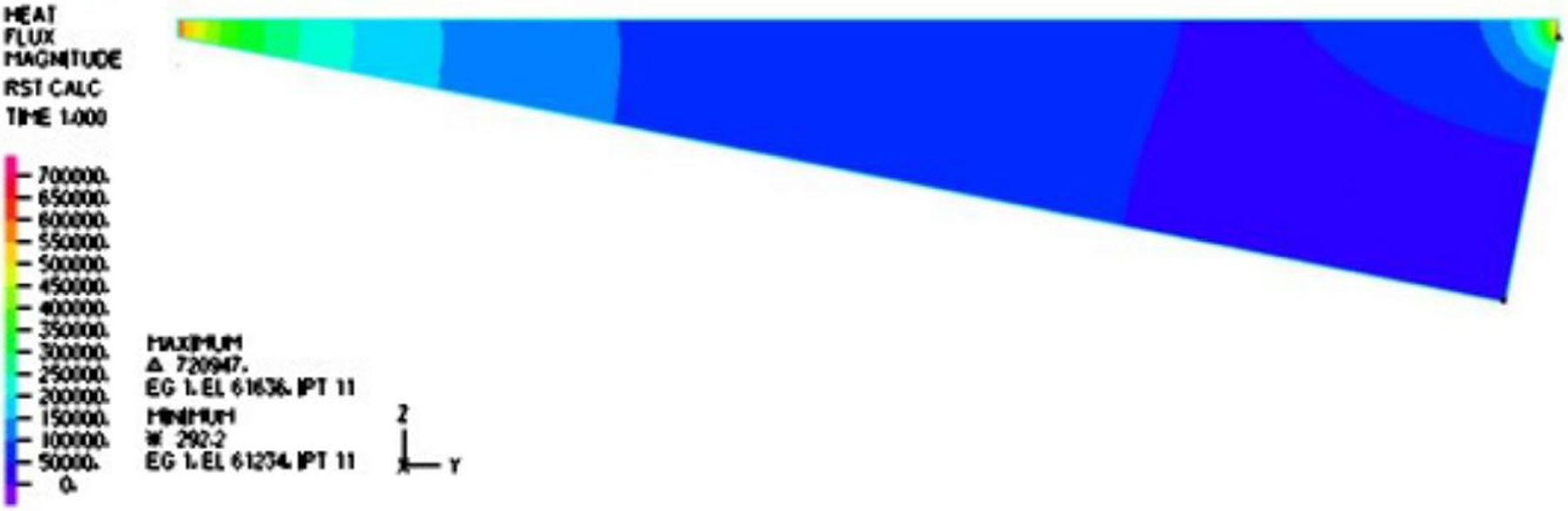
Heat flux distribution within the film for a typical period.

**Figure 5.**
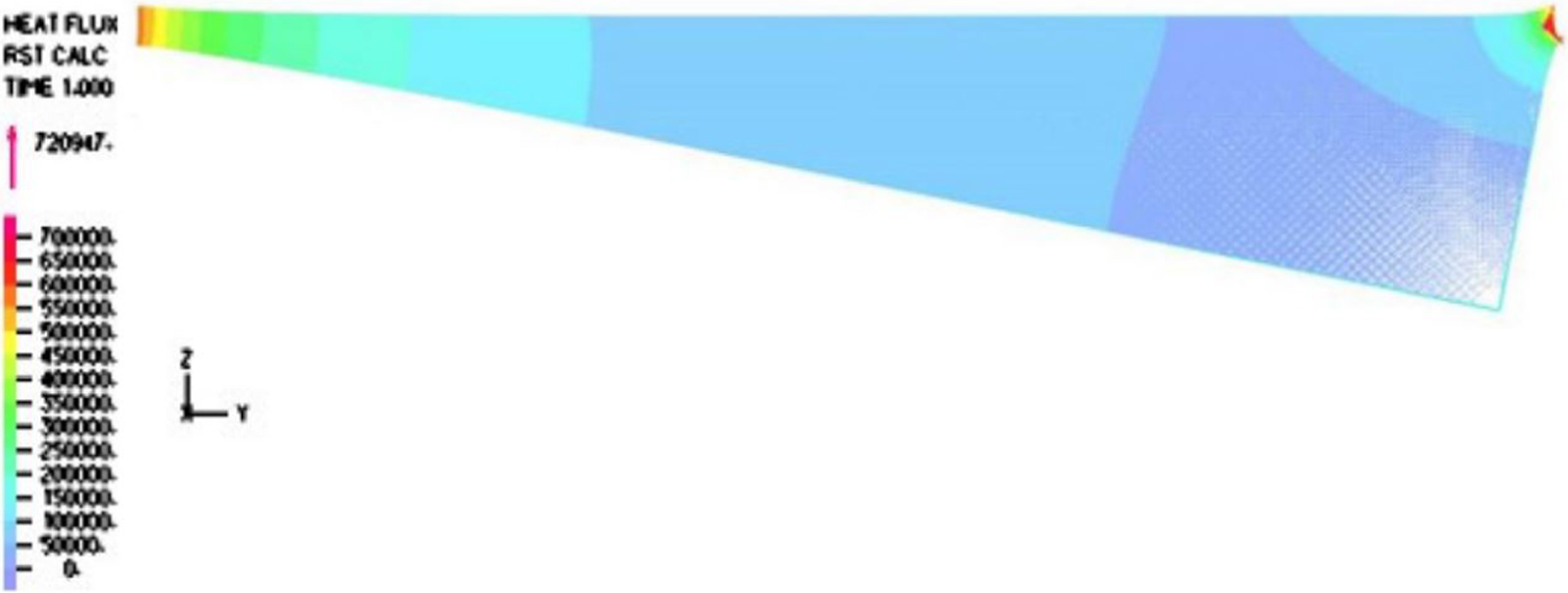
Heat flux vector distribution within the film for a typical period.

The temperature distribution within the vapor film is captured in Figure 3. It is noted that inside the cavity with the aspect ratio of interest to us, the temperature gradient was normal to the top surface, which is confirmed by the vector fields and magnitude distribution of the heat flux in Figures 4 and 5. Furthermore, the temperature gradient was among the highest within the vapor film or cavity.

**Figure 6.**
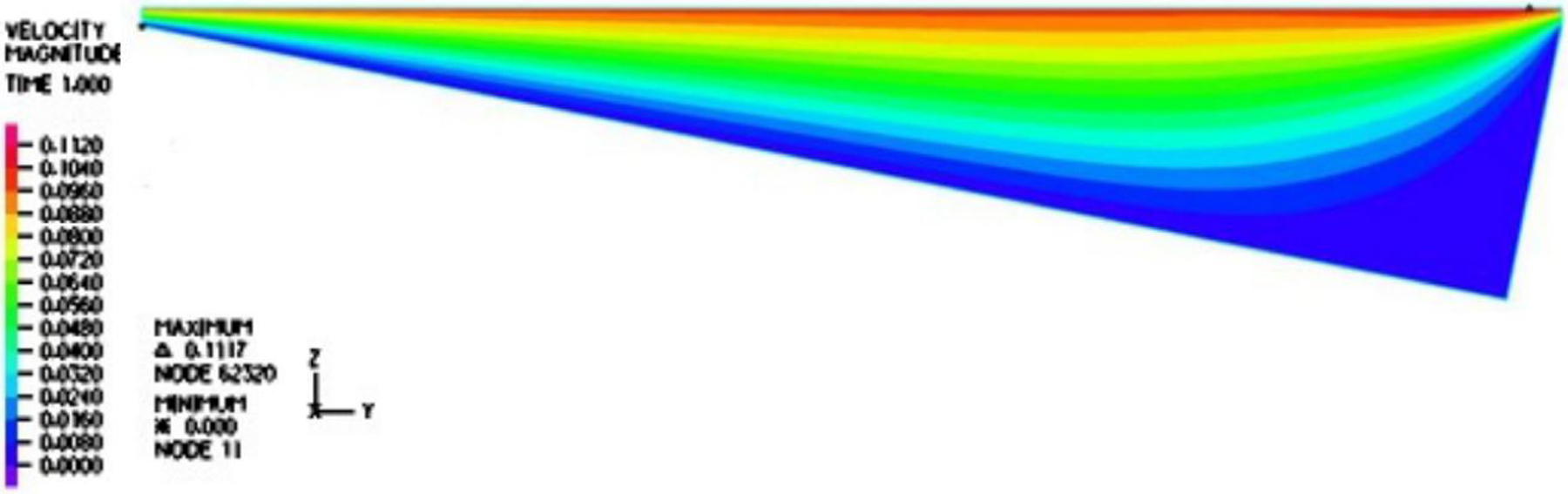
Horizontal velocity distribution within the film for a typical period.

**Figure 7.**
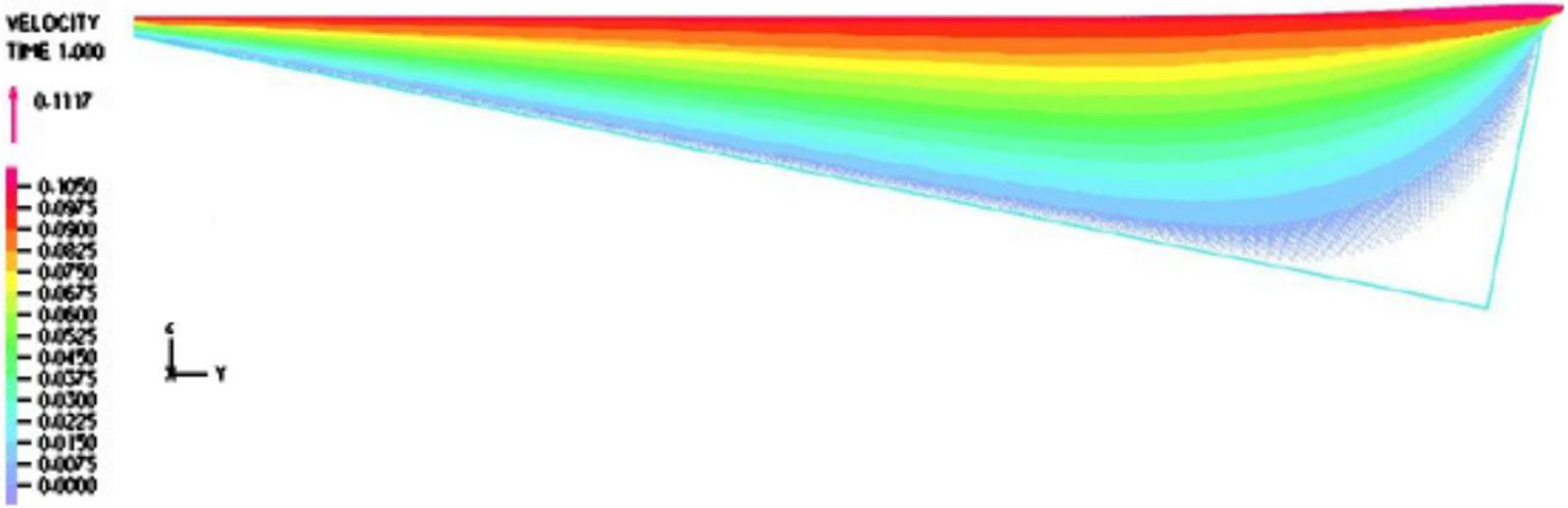
Velocity vector field distribution within the film for a typical period.

**Figure 8.**
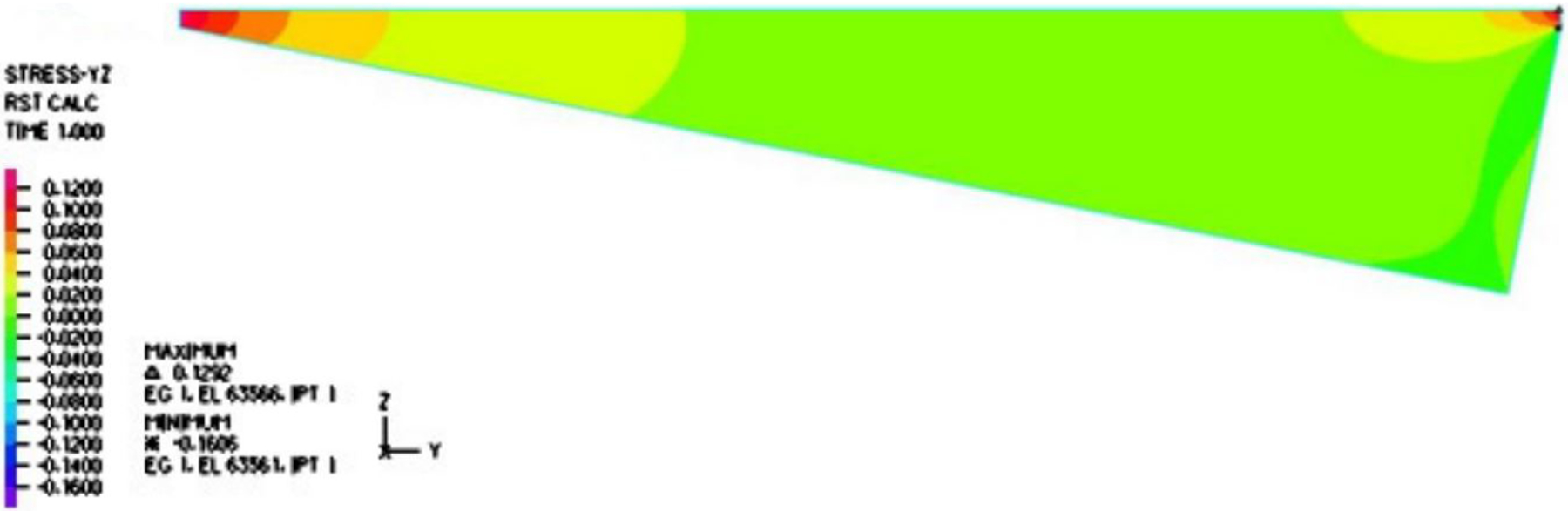
Shear stress distribution on the lower surface of the droplet.

**Figure 9.**
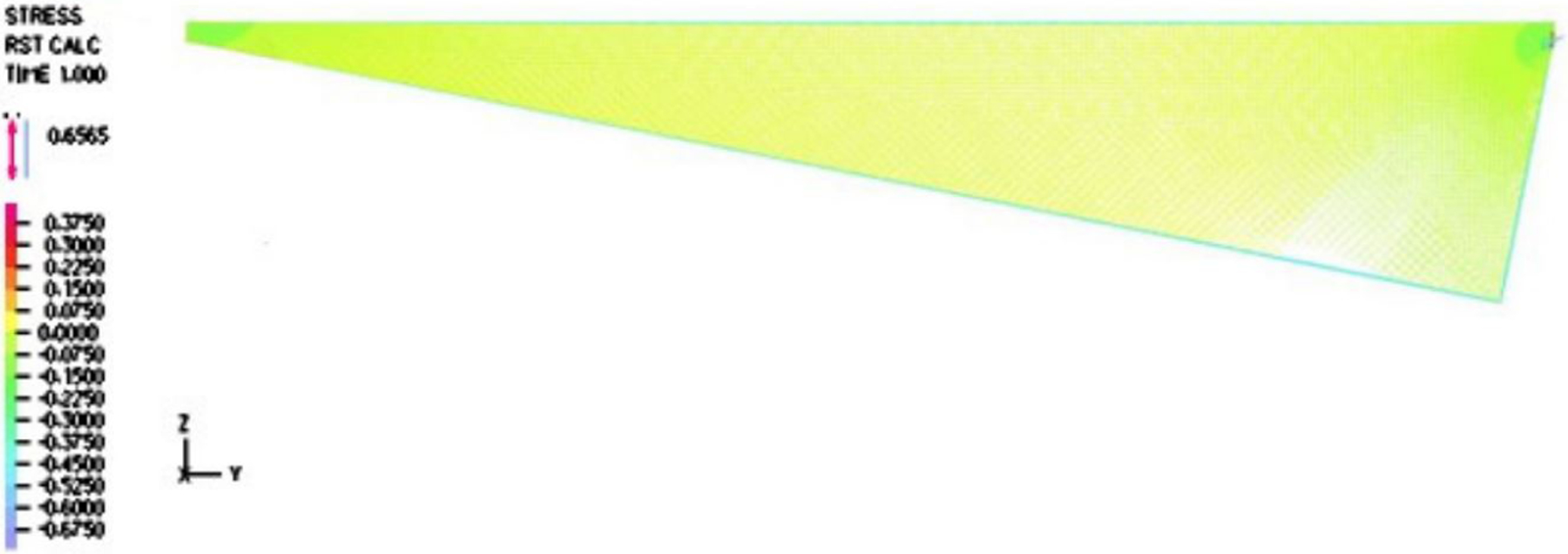
Shear stress vector field distribution within the film for a typical period.

Furthermore, it is evident from Figures 6 and 7 that the velocity within the cavity was a shear-driven Couette flow. In fact, within the period, as shown in Figures 8 and 9, the normalized maximum shear stress τ/V at the moving droplet’s surface merely changed from 0.132 to 1.292 Pa · s/m as depicted in Figure 8, with an average over the period of 0.303 Pa · s/m, which was significantly larger than the one-dimensional estimate as follows:

(9) $$\frac{\tau }{V}≃\mu \frac{1}{d+d_{o}}=0.0828\;Pa\cdot s/m.$$

**Figure 10.**
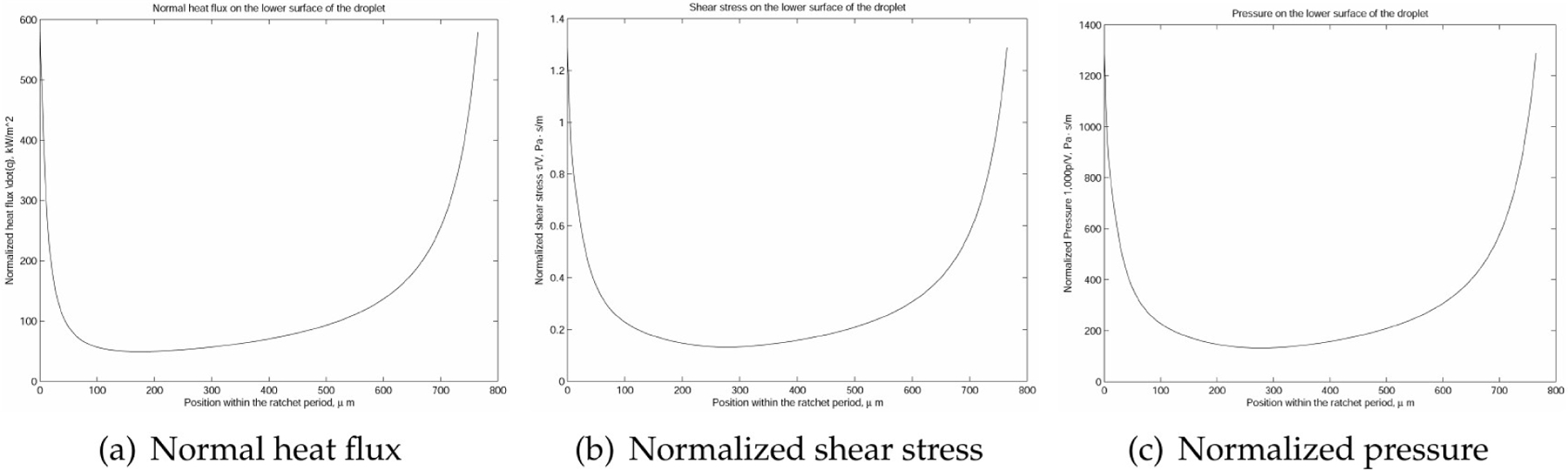
Normal heat flux, normalized shear, and normalized pressure distribution on the lower surface of the droplet.

In this paper, we adopted the average value 0.303 Pa · s/m as the normalized shear stress on the lower surface of the droplet. Likewise, we also compared the simulation results to the simplified thermal conduction calculation. In the vertical heat transfer band plot, it is easy to identify that the normal heat transfer ranged from 49.155 to 584.100 kW/m^2^, as depicted in Figure 10, with an average over the period of 121.903 kW/m^2^, which was on the same magnitude but slightly larger than the high estimate of the one-dimensional simplified model as follows:

(10) $$\dot{q}=k\frac{T_{s}-T_{0}}{d_{0}}≃91.41\;kW/m^{2}.$$

Now, we are ready to handle the linear momentum conservation and the motion of the droplet. Notice that in the simplified models, we first had to use the one-dimensional model to identify the order of magnitude for the thermal conduction, convection, and radiation. However, it was more accurate for us to use the heat flux and the shear stress derived from a more sophisticated two-dimensional model.

The key physics for the propulsion of the droplet is the evaporation-induced loss of linear momentum in the horizontal direction. The governing equation for the droplet can then be expressed as

(11) $$m\frac{dV}{dt}=F_{p}-\left(F_{d}+F_{f}+F_{s}\right),$$

where V stands for the droplet’s traveling velocity in the horizontal direction, $F_{p}$ is the propulsion force due to evaporation, and the air resistance to the motion of the droplet is quantified as three portions, namely, the form drag due to the shape of the droplet $F_{d}$, the skin friction due to the upper surface of the exposed droplet surface $F_{f}$, and the viscous shear within the vapor film between the lower surface of the droplet and the ratchet surface $F_{s}$.

As illustrated in Figure 10, the contributions of the pressure within the vapor film were not as significant as those of the shear stress and the discharge of vapor from the droplet. Needless to say, we assumed that the droplet was afloat and there was no vertical motion in comparison with the horizontal motion. With the help of the two-dimensional fluid dynamics simulation of the vapor film, we have the heat flux and the viscous shear at the lower surface of the droplet as $\dot{q}$ and τ, respectively. More specifically, we have the following averaged values

(12) $$\begin{array}{ll} \dot{q}=121,903 & \;kW/m^{2},\\ \tau =0.3035\;V & \;Pa\cdot s/m, \end{array}$$

the shear force $F_{s}$ can be simply expressed as $\tau A_{l}$ with $A_{l}$ as the lower surface area of the droplet, whereas the rate of evaporation is measured by the rate of the water vapor mass $\dot{m}$, which is calculated as $\frac{\dot{q}}{h_{fg}}$ with $h_{fg}$ as the latent heat for water evaporation, as well as the discharge vapor velocity $v_{0}$, which is calculated as $\frac{\dot{q}}{\rho_{g}h_{fg}}$ with $\rho_{g}$ as the density of water vapor at the saturation temperature at the atmospheric pressure.

According to Ref. [28], at one atmospheric pressure, $h_{fg}$ equals 2257 kJ/kg and $\rho_{g}$ equals 0.5903 kg/m^3^. Thus, adopting the results from our two-dimensional CFD modeling summarized in Equation (12), we can estimate the rate of the discharging vapor as

(13) $$\dot{m}=\frac{\dot{q}}{L}≃0.09645\;kg/{sm}^{2}.$$

Moreover, using the domain near the ratchet, the discharge vapor velocity can also be estimated as

(14) $$v_{o}=\frac{\dot{q}}{\rho_{g}L}≃0.1688\;\text{m}/\text{s}.$$

Therefore, the propulsion due to the discharge of water vapor projected to the horizontal direction can be evaluated as

(15) $$F_{p}=\rho_{g}v_{0}^{2}Nlw\;\text{sin}\;\beta ,$$

where the width of the droplet is denoted as w, and β is the angle of the incline. Ignoring the form drag and the skin drag of the droplet, the terminal velocity of the droplet balancing only the shear stress of the vapor film cavity can be simply calculated as

(16) $$V≃\frac{\rho_{g}v_{o}^{2}}{0.3035}\frac{d}{p}=0.011\;m/s,$$

which yields a simple estimate for the terminal velocity based on both analytical and computational approaches.

This simplified model based on both analytical and computational methods yields a promising result close to the experimental observation. However, more accurate models based on physics can also be proposed. Firstly, as indicated in Figures 4 and 5, the heat flux around the corner is the highest. Adopting the slope defined by the incline angle β, the heat flux around the corner area defined within a radius of 400μm, we have the average heat flux as 128.8 kW/m^2^, which is almost two times the value in Equation (12). While the shear stress and pressure distribution with the consideration of the slope around the corner remain roughly the same.

Consequently, Equations (13) and (14) are modified as

(17) $$\dot{m}=\frac{\dot{q}}{L}≃0.1427\;kg/{sm}^{2}.$$

Moreover, the discharge vapor velocity can be estimated as

(18) $$v_{o}=\frac{\dot{q}}{\rho_{g}L}≃0.268\;m/s.$$

Again, if we ignore the form drag and the skin drag of the droplet, the terminal velocity of the droplet balancing only the shear stress of the vapor film cavity can be simply calculated as

(19) $$V≃\frac{\rho_{g}v_{o}^{2}}{0.3035}\frac{d}{p}=0.0274\;m/s,$$

which yields a much closer simple estimate for the terminal velocity based on both analytical and computational approaches.

Finally, we must reiterate here that the effects of convection and radiation as depicted in Equations (6) and (7) constitute only a very small portion of the heat transfer. The droplet velocity V is not altered significantly.

In aerodynamics, the form drag and skin friction are quantified by the nondimensional form drag coefficient $C_{d}$ and the skin friction drag coefficient $C_{f}$ as well as the dynamic pressure $\frac{1}{2}\rho V^{2}$. Note that the vapor density $\rho_{g}$ is abbreviated as ρ.

Assume that for the duration of observation, the mass loss is insignificant and as a result the radius of the droplet R remains unchanged. Therefore, the final governing dynamic equation for the translational motion of the droplet can be expressed as

(20) $$m\frac{dV}{dt}=\rho v_{o}^{2}A_{l}-\left(\frac{1}{2}\rho V^{2}C_{d}A_{d}+\frac{1}{2}\rho V^{2}C_{f}A_{u}+0.3035VA_{l}\right),$$

where $A_{u},\;A_{l}$, and $A_{d}$ represent the upper-side surface area, the lower-side surface area, and the middle-section cross-section area of the droplet, respectively, and $\frac{1}{2}\rho V^{2}C_{d}A_{d}$ is the form drag $F_{d},\frac{1}{2}\rho V^{2}C_{f}A_{u}$ is the friction drag $F_{f}$ at the upper surface of the droplet, and $0.3035VA_{l}$ is the viscous shear force $F_{s}$ within the vapor film, or the lower droplet surface as depicted in Equation (11).

In this paper, because the lateral shape of the droplet with an approximate volume of 5 microliter is supposed to be close to a spherical shape instead of a pancake shape [25], we approximated $A_{u},\;A_{l}$, and $A_{d}$ as ${2\pi R}^{2},\;{2\pi R}^{2}$, and ${\pi R}^{2}$, respectively. Consequently, we can estimate the terminal velocity using the right-hand side of Equation (20)

(21) $$\rho v_{o}^{2}A_{l}-\left(\frac{1}{2}\rho V^{2}C_{d}A_{d}+\frac{1}{2}\rho V^{2}C_{f}A_{u}+0.3035VA_{l}\right)=0.$$

Notice that the shear stress acting on the droplet as well as the thrust due to evaporation introduces a torque on the droplet. As result, the droplet tends to rotate or tumble as it moves along the horizontal axis. This rotational motion as reported in Refs. [29,30] along with flow instabilities as discussed in Ref. [31] will be studied in a subsequent paper.

## 3. Conclusions

We demonstrated a simplified, two-dimensional CFD heat transfer model of the film-boiling droplet motion on a submillimeter-scale ratchet when the water vapor thickness was less than 100 μm. It was found that the influence of convection and radiation heat transfer to droplet mobility was almost negligible. Our unique single-cavity-based geometric model was very accurate to estimate the experimentally observed terminal velocities of the droplet. Finally, the thermal-conduction-dominant shear viscous model revealed the possible contribution of the self-rotating and tumbling motion of the droplet to the propulsion.

## Data Availability

The original contributions presented in the study are included in the article, further inquiries can be directed to the corresponding author.
